# Use of U-Net Convolutional Neural Networks for Automated Segmentation of Fecal Material for Objective Evaluation of Bowel Preparation Quality in Colonoscopy

**DOI:** 10.3390/diagnostics12030613

**Published:** 2022-03-01

**Authors:** Yen-Po Wang, Ying-Chun Jheng, Kuang-Yi Sung, Hung-En Lin, I-Fang Hsin, Ping-Hsien Chen, Yuan-Chia Chu, David Lu, Yuan-Jen Wang, Ming-Chih Hou, Fa-Yauh Lee, Ching-Liang Lu

**Affiliations:** 1 Endoscopy Center for Diagnosis and Treatment, Department of Medicine, Taipei Veterans General Hospital, Taipei 112, Taiwan; ypwang@vghtpe.gov.tw (Y.-P.W.); cycom122@gmail.com (Y.-C.J.); ioudanny520@hotmail.com (K.-Y.S.); scottenx@gmail.com (H.-E.L.); ifhsin@gmail.com (I.-F.H.); u701117@gmail.com (P.-H.C.); david97lu@gmail.com (D.L.); mchou@vghtpe.gov.tw (M.-C.H.); 2 Division of Gastroenterology, Department of Medicine, Taipei Veterans General Hospital, Taipei 112, Taiwan; fylee@vghtpe.gov.tw; 3 Institute of Brain Science, National Yang Ming Chiao Tung University School of Medicine, Taipei 112, Taiwan; 4 Faculty of Medicine, National Yang Ming Chiao Tung University School of Medicine, Taipei 112, Taiwan; yjwang@vghtpe.gov.tw; 5 Department of Medical Research, Taipei Veterans General Hospital, Taipei 112, Taiwan; 6 Information Management Office, Taipei Veterans General Hospital, Taipei 112, Taiwan; xd.yuanchia@gmail.com; 7 Big Data Center, Taipei Veterans General Hospital, Taipei 112, Taiwan; 8 Department of Information Management, National Taipei University of Nursing and Health Sciences, Taipei 112, Taiwan; 9 Healthcare and Management Center, Taipei Veterans General Hospital, Taipei 112, Taiwan

**Keywords:** artificial intelligence, automated segmentation, U-NET, colonoscopy, colonoscopy preparation quality

## Abstract

Background: Adequate bowel cleansing is important for colonoscopy performance evaluation. Current bowel cleansing evaluation scales are subjective, with a wide variation in consistency among physicians and low reported rates of accuracy. We aim to use machine learning to develop a fully automatic segmentation method for the objective evaluation of the adequacy of colon preparation. Methods: Colonoscopy videos were retrieved from a video data cohort and transferred to qualified images, which were randomly divided into training, validation, and verification datasets. The fecal residue was manually segmented. A deep learning model based on the U-Net convolutional network architecture was developed to perform automatic segmentation. The performance of the automatic segmentation was evaluated on the overlap area with the manual segmentation. Results: A total of 10,118 qualified images from 119 videos were obtained. The model averaged 0.3634 s to segmentate one image automatically. The models produced a strong high-overlap area with manual segmentation, with 94.7% ± 0.67% of that area predicted by our AI model, which correlated well with the area measured manually (r = 0.915, *p* < 0.001). The AI system can be applied in real-time qualitatively and quantitatively. Conclusions: We established a fully automatic segmentation method to rapidly and accurately mark the fecal residue-coated mucosa for the objective evaluation of colon preparation.

## 1. Introduction

Colorectal cancer (CRC) is one of the main malignancies affecting humans, accounting for the second and third most common causes of cancer-related death, respectively, in males and females globally [1]. In the Asia–Pacific area, CRC incidence is also increasing rapidly, and CRC was ranked as the most common cancer over 10 years in Taiwan [1,2]. Colonoscopy is used to image the mucosa of the entire colon and is an effective method for reducing the CRC burden, since colonoscopy can detect CRC early and be used to remove adenomatous polyps, which can significantly improve CRC survival [3,4]. Despite this fact, interval cancer can sometimes be noted in patients who underwent a CRC surveillance program, which may stem from missed lesions due to an incomplete colonoscopy caused by inadequate bowel preparation [5,6,7,8].

Both the American and European Societies of Gastrointestinal Endoscopy have published guidelines on colon preparation to ensure the quality of bowel preparation during colonoscopy [9,10,11]. Inadequate bowel preparation may lead to repeated colonoscopies, prolonged prospective procedure time, increased operative risk, and rising medical costs [12]. Currently, there are three main validated bowel preparation scoring systems for evaluating the quality of colonoscopy preparation, including the Aronchick Scale, the Ottawa Bowel Preparation Scale (OBPS), and the Boston Bowel Preparation Score (BBPS) [13,14,15]. The Aronchick Scale and OBPS evaluate colon preparation before washing and suctioning, while the BBPS evaluates it afterwards [13,14,15]. OBPS also subjectively evaluates the amount of washing and suctioning required to achieve optimal visualization. In addition, the grading system, and the segments used to evaluate preparation (from the whole colon to five divided segments), are also different between the three systems [16,17]. The main concern with these scoring systems is that these scales depend on subjective evaluations to grade bowel cleanliness, which suffer from opinion-related bias [18,19]. That is to say, the inter-observer reliability, measured by intraclass correlations (ICC) or kappa coefficients, would be the major concern of these scales. For example, the Aronchick Scale showed a fair-to-substantial ICC of 0.31–0.76. The ICC of OBPS seems good at 0.94, but this was actually the result from a small-scale study on just a single gastroenterologist and a staff fellow for 97 colonoscopies. In addition, OBPS showed a fair agreement between nurses and physicians with a Pearson’s *r* = 0.60 [20]. The reliability of BBPS is more frequently studied with a fair weighted kappa of 0.67 to 0.78. Among the three scales, the BBPS is the most thoroughly validated and is the most recommended one for use in a clinical setting [18]. Generally, the application of these three scales is time-consuming and requires detailed assessments and documentations. Therefore, in prospectively collected data from a large national endoscopic consortium, the proper application of these scales is rare; only about 11% of doctors in the United States thoroughly evaluate and document the suggested BBPS in clinical practice [21].

In recent years, with the application of artificial intelligence (AI), computer-aided detection and diagnosis software systems have been developed to help endoscopists enhance and characterize polyps during colonoscopy [22,23,24,25]. AI and machine learning techniques have also emerged to evaluate the quality of bowel preparation. Two previous studies explored the evaluation of bowel cleanliness in capsule endoscopy and colonoscopy [26,27]. These applied AI to classify bowel cleanliness based on experts’ subjective grading. With this approach, human factors can still lead to potential bias in scoring due to the fair interobserver reliability of the grading scales used in these reports (capsule endoscopy, ICC = 0.37–0.66; colonoscopy, weighted kappa of 0.67–0.78 with BBPS). In our current study, we used a completely different approach by using a segmentation method to precisely label fecal material in the training dataset. With this method, we attempted to develop a fully automatic segmentation method through the application of convolutional neural networks (CNNs) to mark the mucosal area coated with fecal material using prospectively collected colonoscopy video imaging data. The proposed model can be a useful and novel tool for objectively evaluating the quality of colon preparation. To achieve this goal, we used U-Net, an AI architecture that focuses on biological images, as the backbone in the process [28]. The U-Net architecture won the 2015 International Symposium on Biomedical Imaging (ISBI) cell tracking challenge and is often used for brain tumor cutting [29], retinal image segmentation [30,31], endoscopy image segmentation [32,33], and other medical image segmentation tasks [34,35,36].

## 2. Materials and Methods

### 2.1. Data Collection

Endoscopy video and images from Jan 2019 to Feb 2020 were obtained from the Colonoscopy Video Database from the Endoscopy Center of Taipei Veterans General Hospital. The Colonoscopy Video Database was established by patients who were willing to contribute their colonoscopy video and related profiles for clinical study and consists of 520 videos as of February 2020. All the patients signed an informed consent form to contribute their colonoscopy video for clinical study, and a validated questionnaire for enquiring as to the possible factors contributing to the cleanliness of the bowel preparation was distributed to the participants. All patients received standardized bowel preparation with either 2 L of polyethylene glycol solution or BowKlean^®^ powder (containing sodium picosulfate and magnesium oxite, Genovate Biotechnology, Taiwan) before the colonoscopy. Their endoscopy videos were prospectively obtained from the Colonoscopy Video Database from the Endoscopy Center. All colonoscopies were performed by using an Olympus Evis Lucera Elite CV-290 video processor and a high-definition colonoscope CF-HQ 290 or CF-H290 (Olympus Co., Ltd., Tokyo, Japan). The colonoscopy videos were recorded with a resolution of 1920 × 1080. The patients’ individual information was de-identified and stored in the database. The study was approved by the Institutional Review Board of Taipei Veterans General Hospital.

### 2.2. Image Preprocessing

Initially, all videos were transformed into images according to their sampling rate in frames per second (FPS). Unqualified images were filtered out to ensure good image quality. The unqualified images were too blurred or murky to be recognized, low resolution, or in the improper format, or included frames without stool, or full of stool. Extranious information, such as the examination time, patient ID, name, and sex, were removed. These images were randomly divided into training (90% of the total images) and validation (10% of the total images). After establishing the final model, an independent verification dataset was collected from our center in different period to the time for training/validation [37]. The images used in the different datasets (training/validation/verification) were independent at patient level, indicating that the images from the same patients should be attributed to one particular dataset. The training and validation datasets were used to establish AI models, and the verification dataset served to verify the performance of established AI models. In this task, the data augmentation skill was applied to overcome the limitation of the data quantity and reinforce the performance of the AI model. It is worth mentioning that augmentation skill was only applied to the training dataset to enhance the variation in the training image, and measurement (augmentation) was not used in the validation and verification dataset. The augmentation methods included (1) random rotation (randomly rotated images with preservation), (2) random horizontal flip (horizontally flipped images with random radians), (3) random zoom in/out (zoomed in/out images at random scales), and (4) random Gaussian noise (randomly adding Gaussian noise to images).

### 2.3. Image Labeling

**Figure 1:**
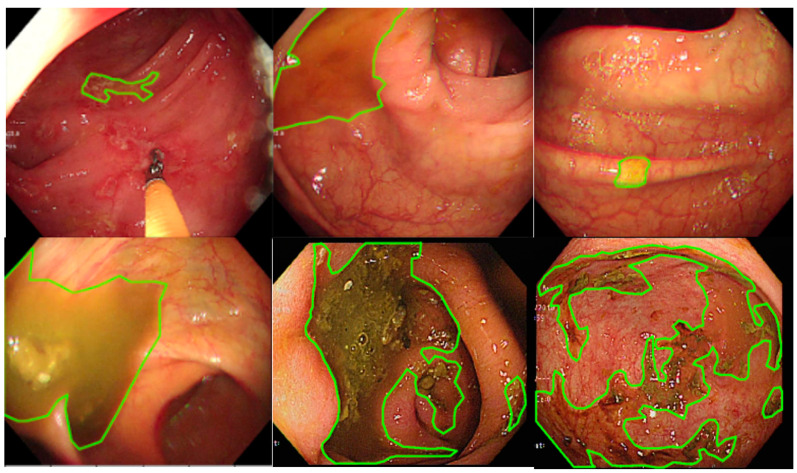
The manual segmentation samples. The figure represents the different types of fecal residues that were annotated and applied in this study.

LabelMe (https://github.com/wkentaro/labelme, accessed on 1 October 2021), an annotation tool for executing image segmentation, is an open-source software and has been widely applied to perform image annotation tasks. The software was installed on a Windows system, and 3 senior endoscopic technicians were trained to perform endoscopy image segmentation labeling (Figure 1). The images show the areas where staining, residual stool, and/or opaque liquid, which influenced the visualization of mucosa, were marked for segmentation [14]. After the annotation, another senior technician rechecked the images to ensure labeling quality. When facing difficulty in image labeling, an experienced endoscopist (Wang YP) was consulted to make the final decision. All images with discernible information were removed and given a random serial number for subsequent model use.

### 2.4. Establishment and Validation of AI Models

**Figure 2:**
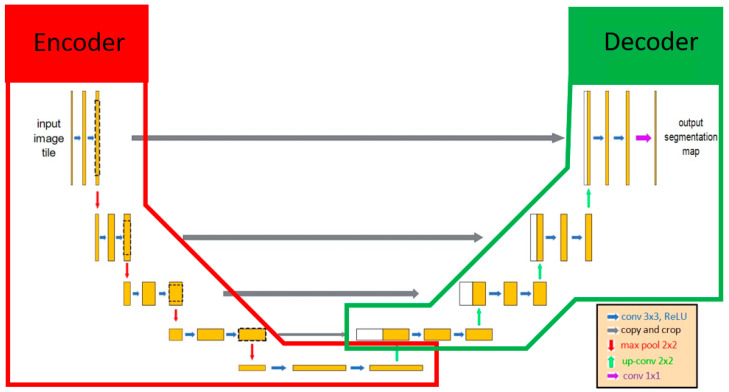
The architecture of U-Net. U-Net contained 2 parts: encoder and decoder. Initially, the input image included features extracted by the encoder, and those features were transmitted to the decoder as the important information for identifying whether each pixel was the target location. The red line and green line indicate the encoder and decoder, respectively, in the U-Net AI model.

U-Net was selected as the main architecture for developing our AI model since U-Net has been deemed valid for medical image recognition [28]. U-Net included 2 parts, the encoder and the decoder. The encoder extracted the important features of the images using the convolution method, and then the decoder applied these features to perform the segmentation task (Figure 2). Various encoders can be selected as the backbone in U-Net architecture for executing feature extraction, such as VGG19, ResNet34, InceptionV3, and EfficientNet-B5 [38]. EfficientNet-B5 was selected in our model because of its better accuracy and lower computational power (Table 1). One of the characteristics of U-Net was that it extracted features that can be transmitted and superimposed on subsequent layers to enhance the information and resolution of neural networks. The output result of U-Net was a probability map, and each pixel of an image had a binary value (0 or 1). The value of the pixels at the target location was segmented as 1, and the other pixel values were assigned to 0. Finally, the result of image segmentation was visualized based on each pixel value.

In U-Net, there still existed some hyperparameters that could be adjusted to enhance the AI performance, such as learning rate, number of epochs, and batch size. During the training process, the validation dataset was used to validate the performance in each trained model. Then, the model with the best performance was saved as the final model. The AI models were trained using Google cloud’s platform with a two-core vCPU, 7.5 GB RAM, and an NVIDIA Tesla K80 GPU. Keras 2.2.4 and TensorFlow 1.6.0 running on CentOS 7 were used for training and validation.

### 2.5. Verification of AI Models and Statistical Analysis

**Figure 3:**
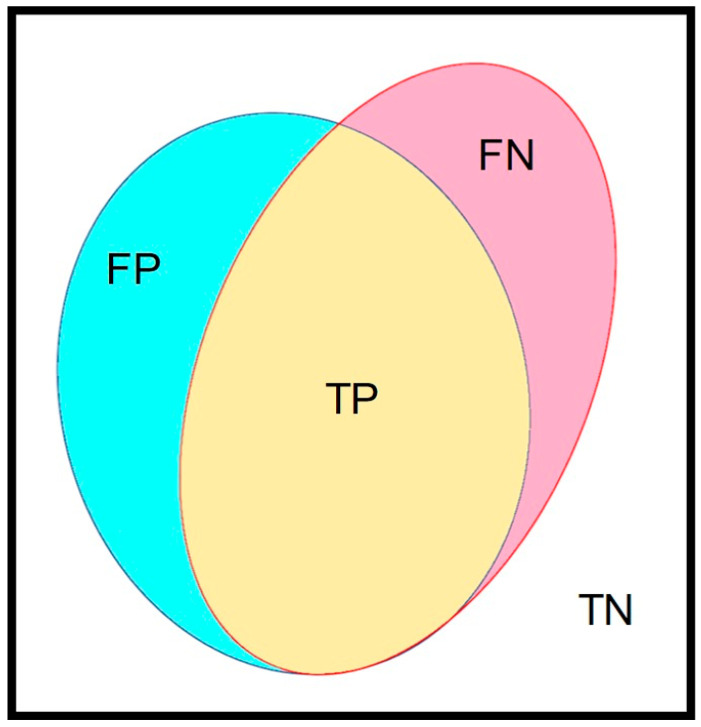
The major parameters in this study. The confusion matrix contained 4 parameters. The yellow area (true positive, TP) represents the intersection area of ground truth and the AI-predicted area. The union of red (false negative, FN) and yellow (true positive, TP) indicate the ground truth area. The blue area (false positive, FP) and yellow area (true positive, TP) indicate the AI-predicted area. The rest of the area out of the union of ground truth and the AI-predicted area was the true negative (TN).

An independent dataset was selected for the verification of the best-established training model. The concept of a confusion matrix was applied to verify the performance of our trained AI model. In our image, the manually marked mucosal area coated by fecal residue was set as the ground truth, which was defined as the union area of false negative (FN) and true positive (TP) (Figure 3). The AI model-predicted area, i.e., the automated segmentation of fecal residue-covered mucosa, involved both the TP and false positive (FP). The intersection area of the ground truth and AI-predicted area was the TP. The area outside of the union of the ground truth and the AI-predicted area was defined as the true negative (TN). Accuracy was calculated as the addition of TP plus TN in proportion to the total mucosal area and was used to represent the performance of our AI model. The defined parameters are given in the following equations:

Intersection over Union (IOU) = TP/(TP + FP + FN)

Accuracy (Acc) = TP + TN/total area       

Predict = (TP + FP)/total area          

GroundTruth = (FN + TP)/total area       

Non_union_percent = TN/total area       

Intersection_percent = TP/total area       

The obtained area in pixels was measured, and all the data are presented as the mean ± S.E.M. The number of pixels in the AI-predicted surface area coated by fecal residue was computed. The proportion of AI-predicted surface area coated by fecal residue against total mucosa area as the octagonal area in the image was also computed and displayed in real time. Pearson correlation and a two-sided *t*-test were used to evaluate the association of the proportion of labelled areas against total area between automatic and manual segmentation. All statistical tests were performed at the α < 0.05 level.

We also selected 3 short videos, each representing poor, good, and excellent preparation, for real-time verification. The final AI model was applied in the video to perform the auto-segmentation of mucosa covered by fecal residue in the video.

## 3. Results

### 3.1. Data Collection

A total of 119 endoscopy videos were collected from 119 patients (mean age: 53.13 years; male/female: 54/65). Successive image frames were then extracted from these videos. After image quality control, a total of 9066 images were selected and randomly divided into two groups, i.e., a training dataset with 8056 images (90% of all images) and a validation dataset with 1010 images (10% of all images). Another dataset for verification containing 1052 images was independently collected from those patients who underwent colonoscopy in a different time period from the training/validation datasets.

### 3.2. The Details of Model Establishment

U-Net, an AI architecture focused on biological image segmentation, was selected as the core architecture in this research. In the training stage, each image was resized to 288 × 288 pixels, the optimizer was set as Adam, the learning rate was set to 1e-4, and the loss function was set as binary cross-entropy. The total training epoch was set to 30, and the batch size was set to four (Table 2).

### 3.3. The Performance of Automatic Segmentation (Results of Model Verification)

The average time required for the model to generate the automatic segmentation of each image was 0.3634 s. The accuracy of our AI model achieved 94.7 ± 0.67% with an IOU of 0.607 ± 0.17. The ground truth (technician-labelled) area of the total area was 14.8 ± 0.43%, while the AI-predicted area was 13.1 ± 0.38% of the total area. The intersection area of the ground truth and AI-predicted area was 11.3 ± 0.36% (fecal material detected by both technician and AI), and the area outside of the union of the ground truth and the AI-predicted area (nonunion area) was 83.4 ± 0.45% of the total measured area (Table 3).

**Figure 4:**
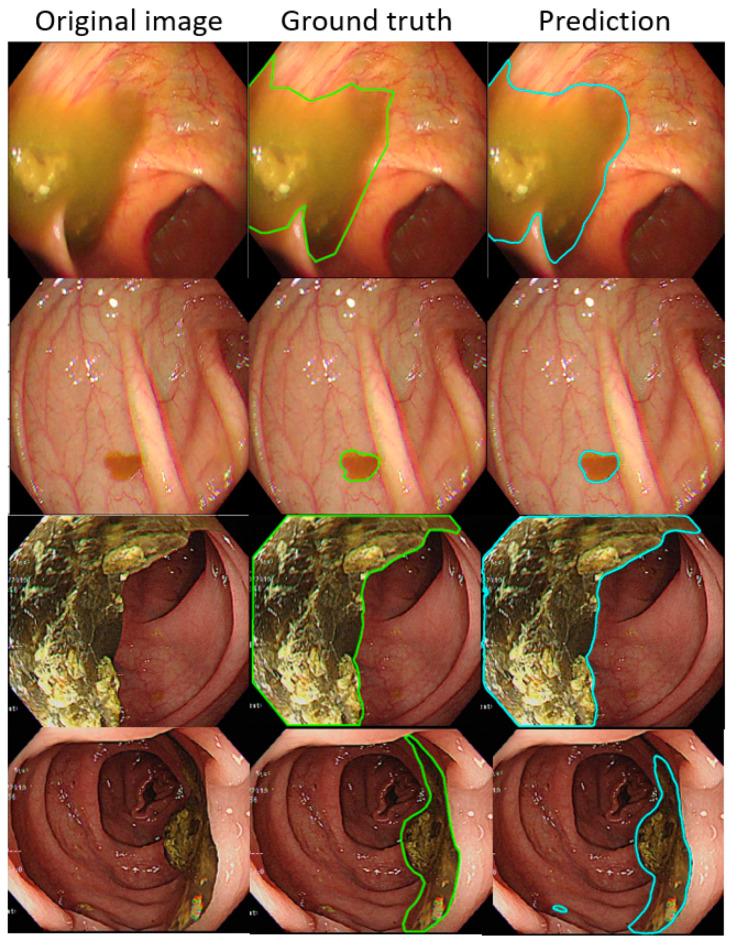
The better annotation example of AI model segmentation. The intersection over union (IOU) of those samples achieved approximately 0.90, meaning that the annotation result of the AI was similar to manual labeling. In those figures, the left, middle, and right columns represent the raw, manually annotated, and AI-annotated images, respectively. The green and blue lines indicate the segmentation labeled by endoscopy technicians and the trained AI model.

**Figure 5:**
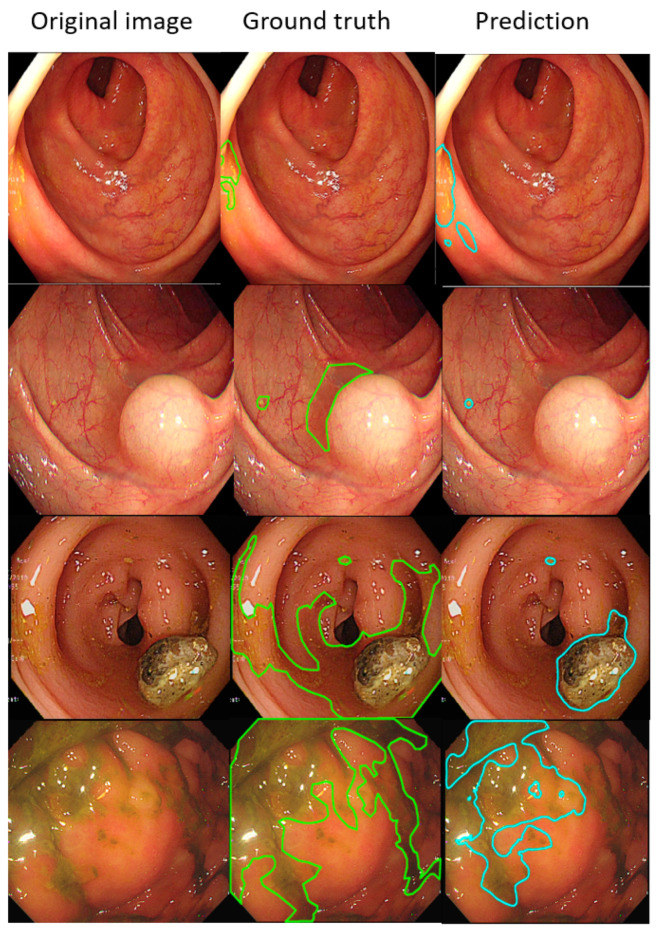
The worse annotation samples of the AI model segment. In each image, the left, middle, and right columns represent the raw, manually annotated, and AI-annotated images, respectively. The green and blue lines indicate the segmentation labeled by endoscopy technicians and the trained AI model. The IOU of these samples was less than 0.5.

Such results suggest that the AI-detected area is 3.5% less than the ground truth (technician-labelled area) (14.8% minus 11.3%), and the rate at which our model misdetected normal mucosa as fecal material is smaller at 1.8% (13.1% minus 11.3%). Example images of the best and worst results of our AI model are displayed in Figure 4 and Figure 5.

**Figure 6:**
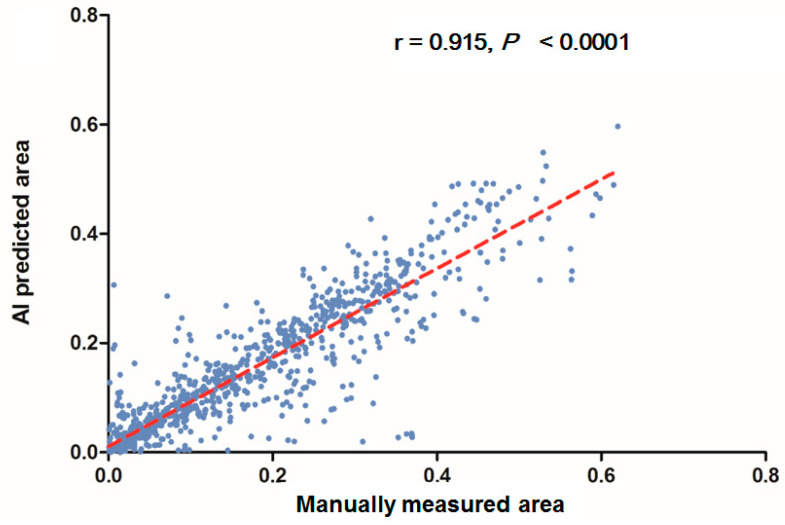
Scatterplots show a comparison of the area produced from manual and automatic segmentation methods.

In each visualized result, the left panel represents the raw image of the verification dataset. The green line in the middle panel indicates the ground truth annotated by endoscopic technicians, and the navy blue line in the right panel represents the result from the AI model prediction. The scatterplots in Figure 6 show that the area segmented manually was highly correlated to the area predicted by the AI (r = 0.915, *p* < 0.001), which suggested the independence of the accuracy with the bowel preparation adequacy. Our AI model was applied in real time in a colonoscopy video with a simultaneous display of the area of auto-segmentation and its percentage of AI-predicted fecal residue-covered mucosa. Example videos of poor, good, and excellent colon cleanliness are shown in Supplementary Videos S1–S3.

## 4. Discussion

In the current study, we used machine learning to evaluate colon preparation using automated segmentation of the mucosal area covered by fecal residue. We demonstrated that this automated segmentation displayed comparable results and high accuracy when compared with manual annotation. To the best of our knowledge, our current article may present the first examples of deep CNN being used for automatically segmenting in the evaluation of the quality of bowel preparation during colonoscopy.

Proper reporting of the preparation quality after colonoscopy is extremely important. Inadequate bowel preparation in colonoscopy will lead to an increased risk of missed lesions, increased procedural time, increased costs, and potentially increased adverse events [21,37]. Furthermore, good preparation scored by the validated bowel preparation scale is associated with an increased polyp detection rate [18]. Currently, there are three main validated bowel preparation scoring systems for evaluating the quality of colonoscopy preparation, including the Aronchick Scale, the OBPS, and the BBPS [13,14,15]. It has been reported that reliability varies between studies and between scales [18,19]. All these scoring systems depend on the endoscopists’ subjective evaluations and are dependent on the raters’ interpretation of visual descriptions. The potential subjective opinion-related bias may lead to a wide difference in grading the adequacy of bowel preparation among physicians, especially in patients with moderate preparation quality that may lead to poor scoring and to a repeat colonoscopy [19]. In this study, we first established an objective evaluation system for bowel preparation by measuring the area of clearly visible mucosa and colon mucosa not clearly visualized due to staining, residual stool, and/or opaque liquid. This machine learning-based scoring system can shift the subjective grading into objectively obtained mucosal areas. The accuracy of this CNN-based model is highly comparable to the manually marked measurement. With this objective measurement system, we may evaluate colon preparation more precisely compared with the subjective grading system. Future studies are mandatory to apply the current AI model to real-world practice and set up an objective threshold for adequate bowel preparation.

Most of the past studies on AI for medical image recognition used retrospectively collected images or video frames to develop their AI models [38,39,40]. In our study, however, we only used video frames to develop our model, which will experience more difficulty achieving a satisfactory result than studies using images or images combined with video frames. This is because video frames are more easily influenced by focus distance, lighting, and vibrations. Therefore, the quality of the frame will often be much lower than that of still images. In some studies, the video verification dataset was significantly lower than the image verification dataset [41,42,43]. Nevertheless, our current model, developed from video frames, displayed satisfactory performance with high auto-segmentation accuracy. Furthermore, after the establishment of our AI model, we verified our model using a dataset that was independent from the dataset used to develop the model. This approach was used to avoid overlap of the training and validation datasets [43].

As shown in the Introduction, we chose U-Net as the core architecture because of its good performance. It may be argued that other architectures may perform better. For example, DeepLab achieved a higher IOU than U-Net in other reports [44,45,46,47,48]. In the decode part, DeepLab would directly quadruple the encoder features as the output result [49], while U-Net obtained the output result by repeating the up-sampling process four times [28]. Hence, U-Net can preserve more low-level features in the final output result. In our case, the fecal material in the image may be relatively small when compared to the entire image. Therefore, we suggest that U-Net may be able to detect more fecal materials, at greater detail, which is more suitable for our purpose. Recent research does suggest that there may be new lightweight encoder networks that may be able to achieve performance on par with the current available encoder with fewer samples while having faster image processing [50]. Future investigations comparing different backbones, especially the lightweight ones, may be necessary to further improve the accuracy and efficiency in AI-assisted fecal material detection during colonoscopy.

Limitations are present in this study. The accuracy of our model when detecting material is high (94%), while the IOU is relatively low (0.61). This may be due to the relatively small annotated area when compared to the entire image, contributing to a high TN in the current model. In addition, our data showed that the area between our model and the ground truth sat in the best line below 0.4 (40% of total area), and it seemed to become more disparate after 0.4 on the scatterplot. Such a result suggests that the current AI model can be less predictive upon poor bowel preparation (images with fecal material more than 40% of total area). The disparate results may be due to the relatively small amount of fecal material in most images used for training. By including more images of poor bowel preparation containing more fecal material during training, we may be able to increase the IOU and improve the accuracy. Concerns may also be raised regarding the accuracy of the manual segmentation as the ground truth, since there are multiple potential variabilities during human annotation. In addition, the cut-off value used to represent the adequacy of bowel preparation and its comparability with the currently validated scoring system are unknown. Additionally, severe bowel inflammation, ulcerations or bleeding may mimic poor colon preparation that influence the evaluation accuracy. Furthermore, we treated the current model as a proof of concept, so the model was established with relatively few images in the validation dataset and lacks the application of k-fold cross-validation. Future studies are mandatory to see whether there are differences among endoscopic technicians on the same images and whether our model falls into the same percentage of errors and deviations in future confirmatory clinical trials.

## 5. Conclusions

In conclusion, we used deep CNN to establish a fully automatic segmentation method to rapidly and accurately mark the mucosal area coated with fecal residue during colonoscopy for the objective evaluation of colon preparation. It is important to evaluate the clinical impact by comparing the application of this novel AI system with the currently available bowel preparation scales.

## Figures and Tables

**Table 1:** Comparison of accuracy using U-Net with different encoders.

Model	Top 1 Accuracy (%)	Top 5 Accuracy (%)	Parameters (M)
VGG19	71.1	89.8	143
Resnet34	73.31	91.4	26
ResNet50+SE	76.86	93.3	28
ResNeXt50	77.15	94.25	25
SENet-154	82.7	96.2	145.8
Inception V3	78	93.9	23.8
DenseNet121	74.5	91.8	8
MobileNet_v2	74.9	92.5	6
EfficientNet-B5	83.3	96.7	30

**Table 2:** The detailed parameters for training the models.

Model	U-Net
Backbone	EfficientNet-B5
Optimizer	Adam
Loss function	binary cross entropy
Learning rate	1e-4
Batch size	4
Total number of epochs run during training	30

**Table 3:** The detailed performance of the final trained models.

	Mean	S.E.M.
IOU	0.607	0.17
Accuracy	0.947	0.0067
Prediction	0.131	0.0038
Ground truth	0.148	0.0043
Intersection area	0.113	0.0036
Nonunion area	0.834	0.0045

IOU = Intersection over union.

## Data Availability

The data presented in this study are available on request from the corresponding author. The data are not publicly available due to Institutional Review Board’s regulation.

## Supplementary Materials

The following supporting information can be downloaded at: https://www.mdpi.com/article/10.3390/diagnostics12030613/s1, Video S1: Example video of poor colon cleanliness, Video S2: Example video of good colon cleanliness, Video S3: Example video of excellent colon cleanliness.

## References

[1] Sung J.J., Lau J.Y., Goh K.L., Leung W.K., Asia Pacific Working Group on Colorectal Cancer (2005). Increasing incidence of colorectal cancer in Asia: Implications for screening. Lancet Oncol..

[2] Chiang C.J., Lo W.C., Yang Y.W., You S.L., Chen C.J., Lai M.S. (2016). Incidence and survival of adult cancer patients in Taiwan, 2002–2012. J. Med. Assoc..

[3] Shaukat A., Mongin S.J., Geisser M.S., Lederle F.A., Bond J.H., Mandel J.S., Church T.R. (2013). Long-term mortality after screening for colorectal cancer. N. Engl. J. Med..

[4] Loberg M., Kalager M., Holme O., Hoff G., Adami H.O., Bretthauer M. (2014). Long-term colorectal-cancer mortality after adenoma removal. N. Engl. J. Med..

[5] Sanduleanu S., Le Clercq C.M., Dekker E., Meijer G.A., Rabeneck L., Rutter M.D., Valori R., Young G.P., Schoen R.E. (2015). Definition and taxonomy of interval colorectal cancers: A proposal for standardising nomenclature. Gut.

[6] Patel S.G., Ahnen D.J. (2014). Prevention of interval colorectal cancers: What every clinician needs to know. Clin. Gastroenterol. Hepatol..

[7] Mitchell R.M., McCallion K., Gardiner K.R., Watson R.G., Collins J.S. (2002). Successful colonoscopy; completion rates and reasons for incompletion. Ulst. Med. J..

[8] Shah H.A., Paszat L.F., Saskin R., Stukel T.A., Rabeneck L. (2007). Factors associated with incomplete colonoscopy: A population-based study. Gastroenterology.

[9] Hassan C., Bretthauer M., Kaminski M.F., Polkowski M., Rembacken B., Saunders B., Benamouzig R., Holme O., Green S., Kuiper T. (2013). Bowel preparation for colonoscopy: European Society of Gastrointestinal Endoscopy (ESGE) guideline. Endoscopy.

[10] Saltzman J.R., Cash B.D., Pasha S.F., Early D.S., Muthusamy V.R., Khashab M.A., Chathadi K.V., Fanelli R.D., Chandrasekhara V., ASGE Standards of Practice Committee (2015). Bowel preparation before colonoscopy. Gastrointest. Endosc..

[11] Rex D.K., Schoenfeld P.S., Cohen J., Pike I.M., Adler D.G., Fennerty M.B., Lieb J.G., Park W.G., Rizk M.K., Sawhney M.S. (2015). Quality indicators for colonoscopy. Gastrointest. Endosc..

[12] Lieberman D.A., Rex D.K., Winawer S.J., Giardiello F.M., Johnson D.A., Levin T.R. (2012). Guidelines for colonoscopy surveillance after screening and polypectomy: A consensus update by the US Multi-Society Task Force on Colorectal Cancer. Gastroenterology.

[13] Aronchick C.A., Lipshutz W.H., Wright S.H., DuFrayne F., Bergman G. (1999). Validation of an instrument to assess colon cleansing. Am. J. Gastroenterol..

[14] Lai E.J., Calderwood A.H., Doros G., Fix O.K., Jacobson B.C. (2009). The Boston bowel preparation scale: A valid and reliable instrument for colonoscopy-oriented research. Gastrointest. Endosc..

[15] Calderwood A.H., Jacobson B.C. (2010). Comprehensive validation of the Boston Bowel Preparation Scale. Gastrointest. Endosc..

[16] Johnson D.A., Barkun A.N., Cohen L.B., Dominitz J.A., Kaltenbach T., Martel M., Robertson D.J., Boland C.R., Giardello F.M., Lieberman D.A. (2014). Optimizing adequacy of bowel cleansing for colonoscopy: Recommendations from the US multi-society task force on colorectal cancer. Gastroenterology.

[17] Kastenberg D., Bertiger G., Brogadir S. (2018). Bowel preparation quality scales for colonoscopy. World J. Gastroenterol..

[18] Parmar R., Martel M., Rostom A., Barkun A.N. (2016). Validated Scales for Colon Cleansing: A Systematic Review. Am. J. Gastroenterol..

[19] Heron V., Martel M., Bessissow T., Chen Y.I., Desilets E., Dube C., Lu Y., Menard C., McNabb-Baltar J., Parmar R. (2019). Comparison of the Boston Bowel Preparation Scale with an Auditable Application of the US Multi-Society Task Force Guidelines. J. Can. Assoc. Gastroenterol..

[20] Martinato M., Krankovic I., Caccaro R., Scacchi M., Cesaro R., Marzari F., Colombara F., Compagno D., Judet S., Sturniolo G. (2013). P.15.8 Assessment of boewel preparation for colonoscopy: Comparison between different tools and different healthcare professionals. Dig. Liver Dis..

[21] Kluge M.A., Williams J.L., Wu C.K., Jacobson B.C., Schroy P.C., Lieberman D.A., Calderwood A.H. (2018). Inadequate Boston Bowel Preparation Scale scores predict the risk of missed neoplasia on the next colonoscopy. Gastrointest. Endosc..

[22] Kudo S.E., Misawa M., Mori Y., Hotta K., Ohtsuka K., Ikematsu H., Saito Y., Takeda K., Nakamura H., Ichimasa K. (2019). Artificial Intelligence-assisted System Improves Endoscopic Identification of Colorectal Neoplasms. Clin. Gastroenterol. Hepatol..

[23] Gong D., Wu L., Zhang J., Mu G., Shen L., Liu J., Wang Z., Zhou W., An P., Huang X. (2020). Detection of colorectal adenomas with a real-time computer-aided system (ENDOANGEL): A randomised controlled study. Lancet Gastroenterol. Hepatol..

[24] Chen P.J., Lin M.C., Lai M.J., Lin J.C., Lu H.H., Tseng V.S. (2018). Accurate Classification of Diminutive Colorectal Polyps Using Computer-Aided Analysis. Gastroenterology.

[25] Byrne M.F., Chapados N., Soudan F., Oertel C., Linares Perez M., Kelly R., Iqbal N., Chandelier F., Rex D.K. (2019). Real-time differentiation of adenomatous and hyperplastic diminutive colorectal polyps during analysis of unaltered videos of standard colonoscopy using a deep learning model. Gut.

[26] Buijs M.M., Ramezani M.H., Herp J., Kroijer R., Kobaek-Larsen M., Baatrup G., Nadimi E.S. (2018). Assessment of bowel cleansing quality in colon capsule endoscopy using machine learning: A pilot study. Endosc. Int. Open.

[27] Zhou J., Wu L., Wan X., Shen L., Liu J., Zhang J., Jiang X., Wang Z., Yu S., Kang J. (2020). A novel artificial intelligence system for the assessment of bowel preparation (with video). Gastrointest. Endosc..

[28] Ronneberger O., Fischer P., Brox T. (2015). U-Net: Convolutional Networks for Biomedical Image Segmentation.

[29] Dong H., Yang G., Liu F., Mo Y., Guo Y. (2017). Automatic Brain Tumor Detection and Segmentation Using U-Net Based Fully Convolutional Networks. Medical Image Understanding and Analysis.

[30] De Fauw J., Ledsam J.R., Romera-Paredes B., Nikolov S., Tomasev N., Blackwell S., Askham H., Glorot X., O’Donoghue B., Visentin D. (2018). Clinically applicable deep learning for diagnosis and referral in retinal disease. Nat. Med..

[31] Chiu S.J., Li X.T., Nicholas P., Toth C.A., Izatt J.A., Farsiu S. (2010). Automatic segmentation of seven retinal layers in SDOCT images congruent with expert manual segmentation. Opt. Express.

[32] Laves M.-H., Bicker J., Kahrs L.A., Ortmaier T. (2019). A dataset of laryngeal endoscopic images with comparative study on convolution neural network-based semantic segmentation. Int. J. Comput. Assist. Radiol. Surg..

[33] De Groof A.J., Struyvenberg M.R., van der Putten J., van der Sommen F., Fockens K.N., Curvers W.L., Zinger S., Pouw R.E., Coron E., Baldaque-Silva F. (2020). Deep-Learning System Detects Neoplasia in Patients with Barrett’s Esophagus with Higher Accuracy than Endoscopists in a Multistep Training and Validation Study with Benchmarking. Gastroenterology.

[34] Zafar K., Gilani S.O., Waris A., Ahmed A., Jamil M., Khan M.N., Sohail Kashif A. (2020). Skin Lesion Segmentation from Dermoscopic Images Using Convolutional Neural Network. Sensors.

[35] Bui T.D., Wang L., Chen J., Lin W., Li G., Shen D. (2019). Multi-task Learning for Neonatal Brain Segmentation Using 3D Dense-Unet with Dense Attention Guided by Geodesic Distance. Domain Adaptation and Representation Transfer and Medical Image Learning with Less Labels and Imperfect Data: First MICCAI Workshop, DART 2019, and first International Work.

[36] Gadosey P.K., Li Y., Adjei Agyekum E., Zhang T., Liu Z., Yamak P.T., Essaf F. (2020). SD-UNet: Stripping Down U-Net for Segmentation of Biomedical Images on Platforms with Low Computational Budgets. Diagnostics.

[37] Clark B.T., Protiva P., Nagar A., Imaeda A., Ciarleglio M.M., Deng Y., Laine L. (2016). Quantification of Adequate Bowel Preparation for Screening or Surveillance Colonoscopy in Men. Gastroenterology.

[38] Gulshan V., Peng L., Coram M., Stumpe M.C., Wu D., Narayanaswamy A., Venugopalan S., Widner K., Madams T., Cuadros J. (2016). Development and Validation of a Deep Learning Algorithm for Detection of Diabetic Retinopathy in Retinal Fundus Photographs. JAMA.

[39] Misawa M., Kudo S.E., Mori Y., Cho T., Kataoka S., Yamauchi A., Ogawa Y., Maeda Y., Takeda K., Ichimasa K. (2018). Artificial Intelligence-Assisted Polyp Detection for Colonoscopy: Initial Experience. Gastroenterology.

[40] Hwang D.-K., Hsu C.-C., Chang K.-J., Chao D., Sun C.-H., Jheng Y.-C., Yarmishyn A.A., Wu J.-C., Tsai C.-Y., Wang M.-L. (2019). Artificial intelligence-based decision-making for age-related macular degeneration. Theranostics.

[41] Fernandez-Esparrach G., Bernal J., Lopez-Ceron M., Cordova H., Sanchez-Montes C., Rodriguez de Miguel C., Sanchez F.J. (2016). Exploring the clinical potential of an automatic colonic polyp detection method based on the creation of energy maps. Endoscopy.

[42] Wang Y., Tavanapong W., Wong J., Oh J.H., de Groen P.C. (2015). Polyp-Alert: Near real-time feedback during colonoscopy. Comput. Methods Programs Biomed..

[43] Wang P., Xiao X., Glissen Brown J.R., Berzin T.M., Tu M., Xiong F., Hu X., Liu P., Song Y., Zhang D. (2018). Development and validation of a deep-learning algorithm for the detection of polyps during colonoscopy. Nat. Biomed. Eng..

[44] Jiang Y., Xiao C., Li L., Chen X., Shen L., Han H. An Effective Encoder-Decoder Network for Neural Cell Bodies and Cell Nucleus Segmentation of EM Images. Proceedings of the 41st Annual International Conference of the IEEE Engineering in Medicine and Biology Society (EMBC).

[45] El-Bana S., Al-Kabbany A., Sharkas M. (2020). A Two-Stage Framework for Automated Malignant Pulmonary Nodule Detection in CT Scans. Diagnostics.

[46] Yao X., Yang H., Wu Y., Wu P., Wang B., Zhou X., Wang S. (2019). Land Use Classification of the Deep Convolutional Neural Network Method Reducing the Loss of Spatial Features. Sensors.

[47] Dozen A., Komatsu M., Sakai A., Komatsu R., Shozu K., Machino H., Yasutomi S., Arakaki T., Asada K., Kaneko S. (2020). Image Segmentation of the Ventricular Septum in Fetal Cardiac Ultrasound Videos Based on Deep Learning Using Time-Series Information. Biomolecules.

[48] Zhang Z., Gao S., Huang Z. (2021). An Automatic Glioma Segmentation System Using a Multilevel Attention Pyramid Scene Parsing Network. Curr. Med. Imaging.

[49] Chen L.-C., Zhu Y., Papandreou G., Schroff F., Adam H. Encoder-Decoder with Atrous Separable Convolution for Semantic Image Segmentation. Proceedings of the Computer Vision—ECCV 2018.

[50] Jeon Y., Watanabe A., Hagiwara S., Yoshino K., Yoshioka H., Quek S.T., Feng M. (2021). Interpretable and Lightweight 3-D Deep Learning Model For Automated ACL Diagnosis. IEEE J. Biomed. Health Inform..

